# Perinatal depression screening using smartphone technology: Exploring uptake, engagement and future directions for the MGH Perinatal Depression Scale (MGHPDS)

**DOI:** 10.1371/journal.pone.0257065

**Published:** 2021-09-29

**Authors:** Rachel Vanderkruik, Edwin Raffi, Marlene P. Freeman, Rebecca Wales, Lee Cohen

**Affiliations:** Ammon-Pinizzotto Center for Women’s Mental Health, Massachusetts General Hospital, Boston, MA, United States of America; University of Oslo, NORWAY

## Abstract

Women may experience new-onset or worsening depressive disorders during pregnancy and the postpartum. If untreated, there may be detrimental consequences to the health and wellbeing of the woman and to her baby. There is a need for improved tools and approaches that can be easily and broadly implemented to effectively detect depression during the perinatal period. Early identification of depression during pregnancy is an important first step towards connecting women to treatment and preventing continued depression into the postpartum or beyond. This report provides preliminary findings from a pilot study of a digital screening app for perinatal depression expiring potential for app reach, engagement, and user demographics and mental health symptoms. With mainly passive recruitment efforts, we collected cross-sectional mental health data on over 700 women during the perinatal period, including women across over 30 countries. We report on mean depression scores among women during pregnancy and the postpartum as well as on constructs that are commonly comorbid with depression, including anxiety, sleep dysregulation, and perceived stress. Over half of the women during pregnancy and over 70% of women in the postpartum had a depression score indicative of clinical depression. Future research directions for this work and potential for public health impact are discussed, including longitudinal data collection and analyses of symptomology over time and embedding evidence-based digital therapeutics into the app as a means to increase access to mental health services.

## Introduction

Mood and anxiety disorders are common in women, affecting approximately twice as many women as men [1]. Mood disorders in women can emerge or worsen with pregnancy and postpartum, with the postpartum period presenting a time of increased risk for the onset or recurrence of affective illness [2]. Up to 85% of women experience some type of postpartum mood disturbance, and it can be difficult to predict who is at greatest risk of serious postpartum psychiatric illness and to disseminate information about risk factors [3, 4]. For most women, the symptoms are transient and relatively mild (i.e., postpartum blues); however, 10–15% of women experience a more disabling and persistent form of mood disorder (i.e., postpartum depression) [5–7]. In a widely cited prospective study of postpartum depression, O’Hara and colleagues describe rates of postpartum major and minor depression combined as equal to 10% [6].

Abundant literature suggests that postpartum depression places the mother at risk for recurrent or refractory illness, and there is evidence of long-term negative effects of postpartum depression on child development and behavior [8]. Given the prevalence of this disorder and its potential to cause significant morbidity in both the child and the mother, there is a need for studies to characterize perinatal depression and to identify risk factors in populations at highest risk for this disorder. While it is difficult to predict which women in the general population will develop postpartum depression, certain risk factors have been identified, including recent stressful life events, inadequate social support, and marital discord or dissatisfaction. A personal history of depression increases one’s vulnerability to peripartum and postpartum depression, and depression during pregnancy is one of the most robust risk factors for depression after delivery [9]. Identification of depression in pregnancy may therefore be an important step towards connecting women with treatments to prevent postpartum depression.

One of the key limiting factors in the study of perinatal depression has been the lack of detailed data on symptomology over time. The current standard of care in screening consists of self-administered depression scales, most commonly the Edinburgh Postnatal Depression Scale (EPDS), an instrument initially designed to be given approximately 6–8 weeks postpartum, although is often used more broadly during the perinatal period [10–13]. More granular data, collected remotely on a daily or weekly basis, could better monitor the trajectory of psychopathology in women who are at high risk during pregnancy and into the postpartum [4, 14]. The feasibility of screening for perinatal depression in both developed and low- and middle-income countries has been explored. The ideal instrument, which has psychometric properties with sufficient sensitivity but also specificity to afford scaled use in large populations with limited treatment capacity, has yet to be identified. For example, the previously mentioned Edinburgh Postnatal Depression Scale (EPDS) has been used in both original and abbreviated forms, and results suggest that both long and short versions have good sensitivity, but the specificity frequently does not exceed 75% [15–18].

A systematic review based on studies performed in low- and middle-income countries (LMICs) has shown specificity of screening tools, such as the EPDS, to be highly variable [19]. The study of perinatal mental health on a global scale is also an area warranting further attention; While estimates of the prevalence of perinatal mood disorders have been on the order of 10–15% in developed countries, the estimated prevalence of these disorders in low resource environments has been substantially greater with sparse proportions of those affected being identified or receiving treatment [20–23]. Relevant questions with respect to screening and treating perinatal depression in LMIC have included the extent to which existing primary care staff and community health workers can both screen for psychiatric disorders such as depression and then deliver treatment, as well as whether available screening tools are valid across populations with different cultural and ethnographic characteristics [19, 24]. This is in addition to the stigma linked to asking for mental health provisions, especially for new mothers who often feel guilty or ashamed of being afflicted with a disorder that is out of their control. Improving the specificity of detection measures may support the rationalized allocation of resources in both constrained and less constrained settings, and leveraging big data and technology may help improve such identification of depression and possibly provide connection to virtual sources of screening and support.

The last few years have seen an unprecedented growth in the number, and technological sophistication, of mobile phones worldwide. The ubiquity of mobile phones makes them highly unobtrusive, and given their general presence, they are significantly less likely to interfere with natural psychosocial behaviors than laboratory-based monitoring devices. Mobile phone applications, with their immediate availability and widespread popularity, allow longitudinal and frequent monitoring of mood states in women who are at a high risk for postpartum depression. Patients might also be more likely to divulge sensitive information when asked in a written (or in app) format and comfortable setting (e.g., at home) as opposed to an interview with a clinician. While there has been recent research exploring the use of mobile apps to screen for perinatal mental health [e.g., 25, 26], these studies have had sample sizes of a couple hundred women. Capturing big data on thousands of women will provide us the granular data needed to better identify and treat perinatal depression in high-risk groups of women. Furthermore, an evolution of the use of apps for screening would lend itself to a potentially seamless introduction to electronically delivered referrals and treatment options. Virtual mental health care has been identified as a likely scenario for delivery of at least a proportion of future mental health care and could be utilized to reduce disparities in access to care [27].

To that end, we developed and pilot-tested a mobile phone application that incorporates various measures to assess psychiatric symptomatology, with a focus on depressive symptoms, in pregnancy and the postpartum period. This application, the MGH Perinatal Depression Scale (MGHPDS), will eventually allow us to collect big data on perinatal women and longitudinally assess risk factors that contribute to depression during this period, and will enable us to do so in a minimally invasive manner. The measures that were selected to be included as part of the MGHPDS were informed by research and clinical experience indicating that perceived stress, sleep disruptions, and anxiety drive risk for perinatal depression [28–32], with the ultimate aim of identifying key items from these measures that are robust predictors for perinatal depression. The present article reports on the first version of the MGHPDS which, which we plan to refine prior to future phases of research and testing. Ultimately, a goal of this work is to narrow down questions being asked in future versions of the app, to reduce participant burden, and to streamline screening for perinatal psychiatric disorders. Perinatal anxiety is understudied and thus a secondary aim of the app is to better understand the longitudinal course of perinatal anxiety and clarify its relationship with perinatal depression. In this current article, we report on the findings from a preliminary test of the first version of the MGHPDS to explore potential for reaching perinatal women on a global scale with the app as well as user engagement with the app over time. We also explore demographics and mental health symptom profile among app users. Findings from this initial exploratory pilot study will inform refinement of the app and data collection methods.

## Methods

### Participants

This study was approved by the Institutional Review Board (IRB) of Mass General Brigham. Subjects were recruited from the Departments of Psychiatry, Primary Care, Obstetrics at MGH, and from MGH-affiliated community health centers. Recruitment efforts from within the hospital and affiliated sites included posting of flyers in hospital-approved areas, placement of flyers and brochures in waiting rooms with authorization from the clinic director, and circulation of emails through the All-User system. We also recruited from our website, www.womensmentalhealth.org and its affiliated listserv. Clinicians who inquired about the study were encouraged to tell their patients about the study and suggest that patients contact our research personnel. Subjects were also recruited from the community by posting flyers in communal areas (e.g., community board posting sites) and placing advertisements in community newsletters and newspapers. We also promoted this study during presentations at national meetings for psychiatrists, obstetricians, and family practitioners. Subjects could also join the study if they searched for or came across the applications within Apple or Google application stores. Anyone who wishes to download and use the app is able to do so free of charge.

Inclusion criteria for this preliminary analysis were women who were: 1) pregnant or up to 12 months postpartum, 2) between the ages of 18–45, 3) able to provide informed consent, 4) willing and able to fill out questionnaires about their mood, sleep, anxiety, and stress levels using a mobile phone application for at least one time point. Exclusion criteria consisted of women who were not pregnant or within 12 months of childbirth or did not complete at least one timepoint assessment (participants were included if there was some missing data from the timepoint assessment).

### Procedures

#### Using the MGHPDS

By downloading the MGHPDS, users may enroll in the research study. However, downloading the app does not automatically enroll a user in the study. Only users who provide consent to participate in the study will be enrolled and their responses to questionnaires will be included as study data. Upon downloading the application, subjects are directed to a screen containing an online consent form that details study participation requirements. This consent form, adapted from standard online survey consent forms, details study goals, the nature of data collection, protection of privacy, and the subject’s right to remove her data from the study at any point in time if she so wishes. Contact information for study personnel is provided in the consent form in the event potential subjects have questions about study participation prior to enrolling. After reading through this consent form screen, subjects were provided with a button to click that states “I agree to participate in this research.” If the subject agrees to consent to the study, a copy of the consent form was emailed to subjects for future reference. If a patient declines to consent for research, she may still use the app.

After obtaining consent, subjects were asked to complete a number of questionnaires, including demographic characteristics (i.e., ethnicity, age, etc.), medical history and psychiatric history, cigarette and alcohol use. Data used to quantify mood, anxiety, sleep, and stress levels across pregnancy and the postpartum period were collected using the mobile phone application. Women completed the battery of questionnaires each time they logged in to the application, although the demographics information was only collected at the first time point. The baseline assessment battery takes approximately 10 minutes to complete. The app is programmed to send participants push notifications (if the participant allows in their phone settings) to complete the questionnaires at the following time points: at 24–26 weeks pregnant, 1–2 weeks postpartum, 4–6 weeks postpartum, and 12 weeks postpartum. Although participants could use the app at multiple timepoints, we report on a cross-sectional analysis in this manuscript for participants who completed the assessment battery for an initial assessment.

#### Included measures

The MGHPDS includes the following measures:

*Edinburgh Postnatal Depression Scale (EPDS)*. The primary outcome variable of interest was the EPDS, a 10-item self-report questionnaire developed to identify women who have postpartum depression. A score of ≥12 was used to signal probable depression [15]. The Cronbach’s alpha for this study was 0.90.
*Patient Health Questionnaire-8-item scale (PHQ-8)*. The PHQ-8 is an 8-item self-report questionnaire that assesses mood and depressive symptoms ranging from 0 (not at all) to 4 (nearly every day); it has been identified as a useful depression measure for population-based studies, and a cut point ≥ 10 can be used for defining current depression [33]. The Cronbach’s alpha for this study was 0.88.
*Generalized Anxiety Disorder-7-item scale (GAD-7)*. The GAD-7 is a 7-item self-report questionnaire that assesses anxiety symptoms with a total score for the seven items that can from 0 to 21. When used as a screening tool for anxiety disorders, further evaluation is recommended when the score is 10 or greater [34]. The Cronbach’s alpha for this study was 0.90.
*Insomnia Severity Index (ISI)*. The ISI is a 7-item self-rated questionnaire used to assess the severity of initial, middle, and late insomnia. A 5-point Likert scale is used to rate each item (e.g., 0 = no problem; 4 = very severe problem), yielding a total score ranging from 0 to 28. The total score is interpreted as follows: absence of insomnia (0–7); sub-threshold insomnia (8–14); moderate insomnia (15–21); and severe insomnia (22–28) [35]. The Cronbach’s alpha for this study was 0.89.
*Perceived Stress Scale (PSS)*. The PSS is a 14-item self-report questionnaire that assesses the severity of perceptions of stress; it measures the degree to which situations in one’s life are appraised as stressful, including perceptions of how unpredictable, uncontrollable, and overloaded respondents find their lives. Individual scores on the PSS can range from 0 to 40 with higher scores indicating higher perceived stress [36]. The Cronbach’s alpha for this study was 0.90.

The scoring guidelines used are made accessible to the user by 1) the described scoring guidelines feature accessible within the application, 2) a copy of the consent form, which delineates the guidelines, and is accessible directly through the application, and 3) a copy of the consent form which is emailed to subjects following enrollment. Please see S1 Appendix for the enumerated scoring guidelines for these questionnaires. We used descriptive statistics to summarize the included participants demographic information and average outcome measure values.

## Results

### Participants and utilization of the app

**Fig 1:**
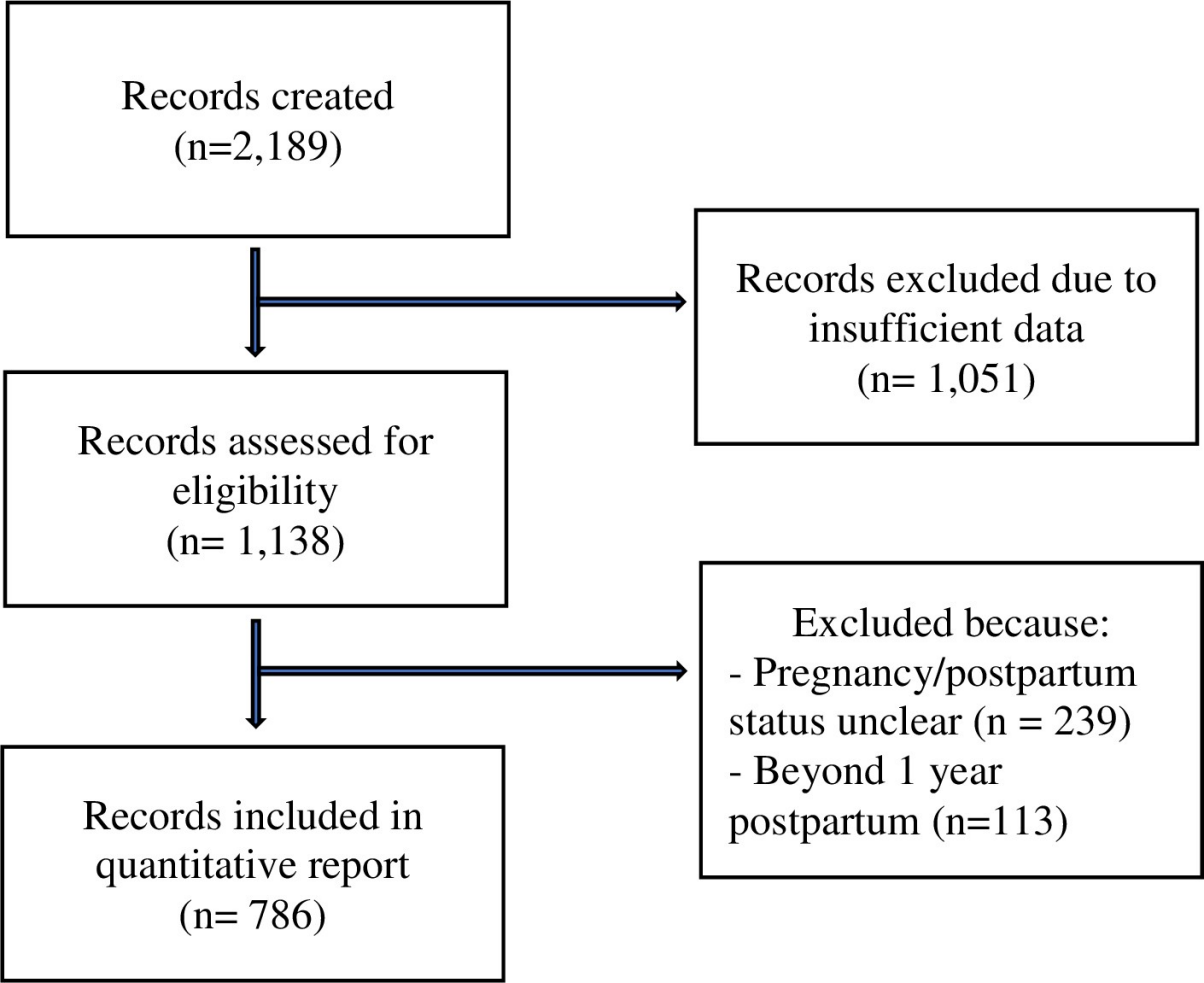
Flow of included participant records.

Between, August 2017 and July 2020, there were 2,189 records (i.e., downloads) of the app. Of those, 1,051 records did not complete one timepoint (missing data allowed) of the assessment battery or did not provide study consent and were thus removed. We removed other records where women reported being outside the perinatal time period or did not provide pregnancy/postpartum status. See Fig 1 for a summary of the 786 participants included in this analysis. See Table 1 for a summary of demographics for the participants included.

**Table 1:** Sample demographics (N = 786).

Age (years), M (SD)	30.30 (5.98)
Race, n (%)	
Caucasian	617 (78.5%)
Asian	40 (5.1%)
African American	32 (4.1%)
American/Indian/Alaska Native	6 (0.8%)
Native Hawaiian/Pacific Islander	1 (0.1%)
Other	56 (7.1%)
More than 1 race selected	18 (2.3%)
Decline to answer	16 (2.0%)
Ethnicity, n (%)	
Non-Hispanic/Non-Latina	649 (82.6%)
Hispanic/Latina	77 (9.8%)
Decline to Answer/Missing Data	60 (7.6%)
Marital status, n (%)	
Single, never married	124 (15.8%)
Married or domestic partnership	637 (81.0%)
Widowed	4 (0.5%)
Divorced	16 (2.0%)
Separated	5 (0.6)
Highest education, n (%)	
No schooling	7 (0.9%)
Some high school, no diploma	22 (2.8%)
High school graduate, diploma	163 (20.7%)
Vocational training	36 (4.6%)
Associate’s Degree	68 (8.7%)
Bachelor’s Degree	230 (29.3%)
Master’s Degree	189 (24.0%)
Doctoral Degree	70 (8.9%)
Missing Data	1 (0.1%)
Pregnancy/Postpartum status	
Pregnant, n (%)	308, (39.2**%**)
Weeks, m (SD)	21.97 (11.15)
Postpartum, n (%)	478, (60.8**%**)
Weeks, m (SD)	16.22 (14.28)
Pregnancy History, n (%)	
>1 prior pregnancy	522 (66.4%)
Never pregnant before	262 (33.3%)
Missing Data	2 (0.3%)
Smoking, n (%)	
Never	563 (71.6%)
Former	175 (22.3%)
Current	47 (6.0%)
Missing Data	2 (0.1%)
Current Alcohol Use, n (%)	
Yes	191 (24.3%)
No	594 (75.6%)
Missing Data	1 (0.1%)

See Table 1 for a summary of demographics for the participants included. Most participants were from the United States of America (n = 433), followed by Canada (n = 46), Australia (n = 10), and the United Kingdom (n = 9). There were 1–3 participants from the following countries: Angola, Anguilla, Belgium, Bermuda, Brazil, Chile, China, Cyprus, Ecuador, Germany, Greece, Iceland, Ireland, Israel, Japan, Mexico, New Zealand, Pakistan, Panama, Philippines, Puerto Rico, Romania, Serbia, Singapore, South Africa, Switzerland, Turkey, United Arab Emirates. There were 245 participants who did not report their country.

### Mental health measures

See Table 2 for a summary of mean scores for the mental health variables of interest by sample overall and broken down by women who were pregnant and women who were within 1 year postpartum. Just over half (51%) of women in pregnancy and 71.7% of women in the postpartum had EPDS scores ≥ 12.

**Table 2:** Mental health outcomes (sample overall, and by pregnancy status).

Outcome Variables	Overall	Pregnant Women	Postpartum Women
	(n = 786)	(n = 308)*	(n = 478)**
**Depression**			
PHQ-8	9.51 (6.03)	8.42 (5.70)	10.21 (6.13)
EPDS	14.01 (6.40)	12.43 (6.60)	15.02 (6.10)
**Anxiety**			
GAD-7	9.22 (5.81)	7.95 (5.70)	10.04 (5.74)
**Sleep**			
ISI	10.29 (6.30)	9.85 (6.40)	10.58 (6.22)
**Perceived Stress**			
PSS	31.31 (10.85)	28.36 (10.54)	33.22 (9.31)

*Notes*: * N ranged from 304 to 308 for these measures due to missing data;

** N ranged from 473 to 478 for these measures due to missing data.

## Discussion and conclusions

This report provides preliminary findings from a pilot study which explored the potential for reaching and engaging perinatal women on a global scale with the MGHPDS app over time. We also explore demographics and mental health symptom profile among app users. There is a need for improved tools and approaches that can be easily and broadly implemented to effectively detect depression during the perinatal period. Early identification of depression during pregnancy is an important first step towards connecting women to treatment and preventing continued depression into the postpartum. Leveraging the power of big data and technology can enable us to fine tune what we know regarding predictors of perinatal depression. Furthermore, the development and dissemination of an easy-to-use screening app could aid with detection of perinatal depression among hard-to-reach patients, such as those living in rural or low-resourced locations, nationally and internationally. In resource-limited settings, the risks of using screening tools with inadequate specificity include overwhelming already stretched resources by generating referrals to treatment of false-positive cases. Improving the specificity of detection measures may thus support the rationalized allocation of resources in both constrained and less constrained settings.

With mainly passive recruitment efforts we collected mental health data on over 700 women during the perinatal period, reaching women across over 30 countries. Such speaks to the promise of digital screening and how, with greater dissemination efforts, we could aim to collect data from a larger and more diverse sample on a global scale. The increased use of digital technology for both identification and treatment across disease states, including mental health disorders, provides an opportunity to develop an abbreviated perinatal depression screening tool with both high sensitivity *and* specificity exceeding what is currently available. The use of mobile cellular phones provides an opportunity to screen very large samples of subjects for perinatal depressive symptoms, as well as commonly comorbid symptoms such as anxiety, sleep dysregulation, and perceived stress. The large sample would naturally allow for powered analysis of symptoms when determining best screening practices. This can then be coupled with real time instrument scoring and electronic data capture, providing an opportunity to refine currently available screening tools and to develop digital platforms that can be used in both developed and developing countries.

From a global perspective, this app could be of great interest to low and middle income (LMIC) countries where resources and perinatal mental health tools and services are limited. To date, use of a mobile phone application to screen for perinatal depression has been demonstrated to be feasible in a low resource environment in South Africa where community health workers administered the EPDS using mobile phones [19]. A 4-item screening tool for symptoms of common mental disorders and suicidality has been developed, validated and found to be effective at identifying pregnant women with symptoms of common mental disorders across commonly spoken languages and cultures in Cape Town, South Africa [37, 38]. A recent review of the literature on digital technology for treating and preventing mental disorders in LMIC concluded that, while continued research is needed, findings across 49 studies were promising and demonstrated potential effectiveness of online, text-messaging, and telephone support interventions [39]. Further research is needed to assess 1) the extent to which existing primary care staff and community health workers can both screen for perinatal psychiatric disorders and then deliver treatment, 2) whether available screening tools are valid across populations with different cultural and ethnographic characteristics, and 3) whether self-reported measures delivered by an app can yield greater engagement in treatment [19, 24]. Clearly, accurate screening and reliable diagnosis of perinatal depression will be an essential first step to the ultimate hurdle, which is to build the bridge from detection to delivery of effective evidence-based treatments to those suffering from depression where substantial mental health service resources are limited or simply do not exist.

The majority of the users reported clinically significant levels of depression (51% pregnant women, 72% postpartum women); although, one likely limitation may be selection bias in the findings in that women who are experiencing more depressive symptoms seek out and more actively engage with the app. However, this also suggests that women experiencing depressive symptoms are seeking support services and informational resources through apps. Thus, there is an opportunity to leverage this app and connect women who screen above a specified threshold to an intervention or resources. Furthermore, detection of women experiencing depression in pregnancy could serve as a means to prevent postpartum depression, if these women are then able to be connected to evidence-based depression treatment. Digital technology, including mobile health (mHealth), has been identified as a promising strategy for increasing access to evidence-based intervention for mental health due to factors such as the constant availability, equity of availability, immediate support, low cost, and fact that digital technology can overcome geographic barriers or regional shortages of mental health providers [40]. A systematic review of the effectiveness of mobile apps for monitoring and management of mental health symptoms or disorders concluded that there is support for the potential of apps to effectively reduce mental health symptoms, yet further robust research is needed to develop and test these evidence-based app programs [41]. Specific to the perinatal population, a recent meta-analysis provides support for the efficacy and acceptability of internet-delivered interventions for perinatal women, and highlights the limited number of interventions utilizing technology specifically for this population, and particularly for mental health symptoms during pregnancy [42]. Given low rates of treatment seeking, preferences for care in non-specialty settings, and limited services targeting depression during pregnancy, directing users of the MGHPDS whose scores indicate possible depression to a digital therapeutic intervention could be a highly impactful service [43].

We have identified several future additional directions for this work utilizing the MGHPDS. First, we aim to increase engagement for longitudinal data collection. Engagement with the app over time was minimal; we report only on cross-sectional data as only a small percentage of participants returned to the app for a second time to fill out measures. We will focus efforts further on user engagement over time for mobile applications to develop features and strategies that would encourage participants to return to the app and complete the measures longitudinally at multiple time points over pregnancy and the postpartum. This would enable us to examine the predictors of depression across time in pregnancy and the postpartum.

Relatedly, we hope to explore more about how to optimize the user experience with the MGHPDS for future versions of the app. We will be adding a brief optional survey in the app to obtain a better understanding of what brought women to the app, what their experiences were with using the app, and what they did with the information and scores provided through the app. This knowledge will enable us to fine tune the app to better meet the needs of women in the perinatal period and to improve their satisfaction with using the app. There is also an opportunity to explore in which types of environments women are more likely to report their symptoms of depression or seek treatment. For example, future research could explore differences in reporting depression symptoms when assessed over an app in the privacy of their home compared to an office and with a healthcare provider. Another area of research could be pertaining to the uptake of treatment when screening is conducted via an app and connected to a digital therapeutic. Furthermore, as the trajectory of perinatal anxiety is understudied, we intend to build upon this work as it relates to screening for anxiety, its longitudinal course, and its relationship with elevated depressive symptoms.

As with any data collected through a widely accessible app or survey, an area of concern is with regards to the quality and validity of the data. We plan to conduct a validation study for this screening tool with a sample of participants where we administer a structured psychiatric diagnostic interview (e.g., using the MINI [44]) that will serve as the gold-standard for depression diagnosis. Ultimately, we aim to leverage “big data” to modify available perinatal depression screening tools by potentially including symptoms such as anxiety, sleep dysregulation, and perceived stress with which perinatal depression is so often associated. Longer term aims will include exploring options for embedding evidence-based digital therapeutics into the app as a means to increase access to mental health services. The use of digital technology with an app that allows for both substantial sample size and statistical power as well as accurate scoring, and ultimately a version of the app which is brief, accurate, and can be easily administered (e.g., by community health workers or non-specialist providers) is a significant step forward for the field.

## Supporting information

S1 Appendix Scoring guidelines. (DOCX)
