# Supplementary material for: Perinatal depression screening using smartphone technology: Exploring uptake, engagement and future directions for the MGH Perinatal Depression Scale (MGHPDS)
Source: PLoS One. 2021 Sep 29;16(9):e0257065. doi: 10.1371/journal.pone.0257065 (PMC8480830; doi:10.1371/journal.pone.0257065)
Supplement: S1 Appendix — (DOCX) [file pone.0257065.s001.docx]

**APPENDIX S1. SCORING GUIDELINES**

Upon completion of a questionnaire, the user will be given the score for the questionnaire, along with the scoring guidelines for each questionnaire. The scoring guidelines to be presented are listed below. These guidelines are taken from the Edinburgh Postnatal Depression Scale for the EPDS, the Patient Health Questionnaire-8-item scale for the PHQ-8, and the Generalized Anxiety Disorder-7-item scale for the GAD-7, and were adapted to match the clinical guidelines for the remaining questionnaires.

*Edinburgh Postnatal Depression Scale (EPDS):*

| Your Score: [##]  Question 10 Score: [#]   - If you scored a 1, 2 or 3 on question 10, PLEASE CALL YOUR HEALTH CARE PROVIDER (OB/Gyn, family doctor, nurse-midwife, psychiatrist, therapist, etc.) OR GO TO THE EMERGENCY ROOM NOW to ensure your own safety and that of your baby. - If your total score is 11 or more, you could be experiencing depression or anxiety. PLEASE CALL YOUR HEALTH CARE PROVIDER (OB/Gyn, family doctor, nurse-midwife, psychiatrist, therapist, etc.) now to keep you and your baby safe. - If your total score is 9-10, we suggest you repeat this test in one week or call your health care provider (OB/Gyn, family doctor, nurse-midwife, psychiatrist, therapist, etc.). - If your total score is 1-8, new mothers often have mood swings that make them cry or get angry easily. Your feelings may be normal. However, if they worsen or continue for more than a week or two, call your health care provider (OB/Gyn, family doctor, nurse-midwife, psychiatrist, therapist, etc.). Being a mother can be a new and stressful experience. Take care of yourself by:   - Getting sleep   - Asking friends and family for help.   - Drinking plenty of fluids.   - Eating a good diet.   - Getting exercise, even if it’s just walking outside. - Regardless of your score, if you have concerns about depression or anxiety, please contact your health care provider.   *Please note: The Edinburgh Postnatal Depression Scale (EPDS) is a screening tool that does not diagnose postpartum depression (PPD) or anxiety.* |
| --- |

*Patient Health Questionnaire-8-item scale (PHQ-8):*

| Your Score: [##]   - If your total score is 10 or more, you could be experiencing depression or anxiety. Please consider calling your health care provider (OB/Gyn, family doctor, nurse-midwife, psychiatrist, therapist, etc.) now to discuss possible follow-up. - If your total score is 5-9, we suggest you repeat this test in two weeks or call your health care provider (OB/Gyn, family doctor, nurse-midwife, psychiatrist, therapist, etc.). - If your total score is 1-4, new mothers often have mood swings that make them cry or get angry easily. Your feelings may be normal. However, if they worsen or continue for more than a week or two, call your health care provider (OB/Gyn, family doctor, nurse-midwife, psychiatrist, therapist, etc.). Being a mother can be a new and stressful experience. Take care of yourself by:   - Getting sleep   - Asking friends and family for help.   - Drinking plenty of fluids.   - Eating a good diet.   - Getting exercise, even if it’s just walking outside. - Regardless of your score, if you have concerns about depression or anxiety, please contact your health care provider.   *Please note: The Patient Health Questionnaire-8-item scale (PHQ-8) is a screening tool that does not diagnose postpartum depression (PPD) or anxiety.* |
| --- |

*Generalized Anxiety Disorder-7-item scale (GAD-7):*

| Your Score: [##]   - Score 15-21: May indicate severe anxiety. - Score 10-14: May indicate moderate anxiety. - Score 5-9: May indicate mild anxiety. - Score 0-4: May indicate none-minimal anxiety. - Please consider calling your healthcare provider (OB/Gyn, family doctor, nurse-midwife, psychiatrist, therapist, etc.) if your symptoms were indicative of possible moderate or severe anxiety. - Regardless of your score, if you have concerns about depression or anxiety, please contact your health care provider.   *Please note: The Generalized Anxiety Disorder-7-item scale (GAD-7) is a screening tool that does not diagnose postpartum depression (PPD) or anxiety.* |
| --- |
